# The impact of STAS on recurrence-free and overall survival in T1-2N0 adenocarcinomas: a single-center retrospective study

**DOI:** 10.1186/s12885-026-16623-w

**Published:** 2026-07-27

**Authors:** Şebnem Dursun, Saliha Yılmaz, Yusuf Kahya, Gökhan Kocaman, Süleyman Gökalp Güneş, Duru Karasoy, Serkan Enön, Serpil Dizbay Sak, Ayten Kayı Cangır

**Affiliations:** 1 Department of Thoracic Surgery, Ankara University Faculty of Medicine, Talatpaşa Blv, İbni Sina Hospital, Altındag, Ankara, Türkiye; 2 Department of Statistics, Hacettepe University Faculty of Science, Beytepe, Ankara, Türkiye; 3 Department of Medical Pathology, Ankara University Faculty of Medicine, Talatpaşa Blv, İbni Sina Hospital, Altındag, Ankara, Türkiye; 4 Ankara University Medical Design Research and Application Center, MEDITAM, Ankara, 06520 Türkiye; 5 Ankara University, Integrated Technologies Research Center, BUTAM, Ankara, 06520 Türkiye

**Keywords:** STAS, Lung adenocarcinoma, Early-stage lung cancer, Recurrence-free survival, Overall survival

## Abstract

**Background:**

Adenocarcinoma (AC) is the leading histopathological type of lung cancer (LC), curative surgery being the cornerstone for the best survival results. However, even in the patients who had curative surgery, the survival outcomes differ. Spread through air spaces (STAS), recognized as a histopathological feature in the 2015 World Health Organization (WHO) Classification, causes poor survival outcomes in lung AC. This study aimed to evaluate the prognostic impact of STAS on recurrence-free survival (RFS) and overall survival (OS) in surgically resected T1-2N0 lung AC, with particular attention to its interaction with tumor size.

**Methods:**

This single center retrospective study evaluates the effect of STAS on RFS and OS in 296 surgically resected T1-2N0 lung AC patients. For further analysis, patients were divided into four subgroups combining their STAS status and tumor size.

**Results:**

STAS presence rate was 37.5%, which was associated with aggressive tumor characteristics, such as non-lepidic predominant subtypes, higher pathological T stage (pT), visceral pleura invasion (VPI), and lymphovascular invasion (LVI). OS and RFS were significantly worse in the STAS-Present (STAS-P) group (*p* = 0.030). Subgroup analysis revealed that STAS-P tumors > 2 cm had the worst outcomes, as expected. Notably, on univariate analysis STAS presence appeared most prognostically decisive in tumors ≤ 2 cm, in which there was no significant difference in survival between STAS-P tumors ≤ 2 cm and STAS-Absent (STAS-A) tumors > 2 cm.

**Conclusions:**

STAS was a significant prognostic factor on univariate analysis but did not retain independent significance after multivariable adjustment. In an exploratory subgroup analysis, STAS-P tumors ≤ 2 cm carried survival outcomes comparable to STAS-A tumors > 2 cm, suggesting STAS may attenuate the prognostic advantage of smaller tumor size. This hypothesis-generating finding warrants prospective validation before informing surgical management.

**Graphical Abstract:**

**Figure:**
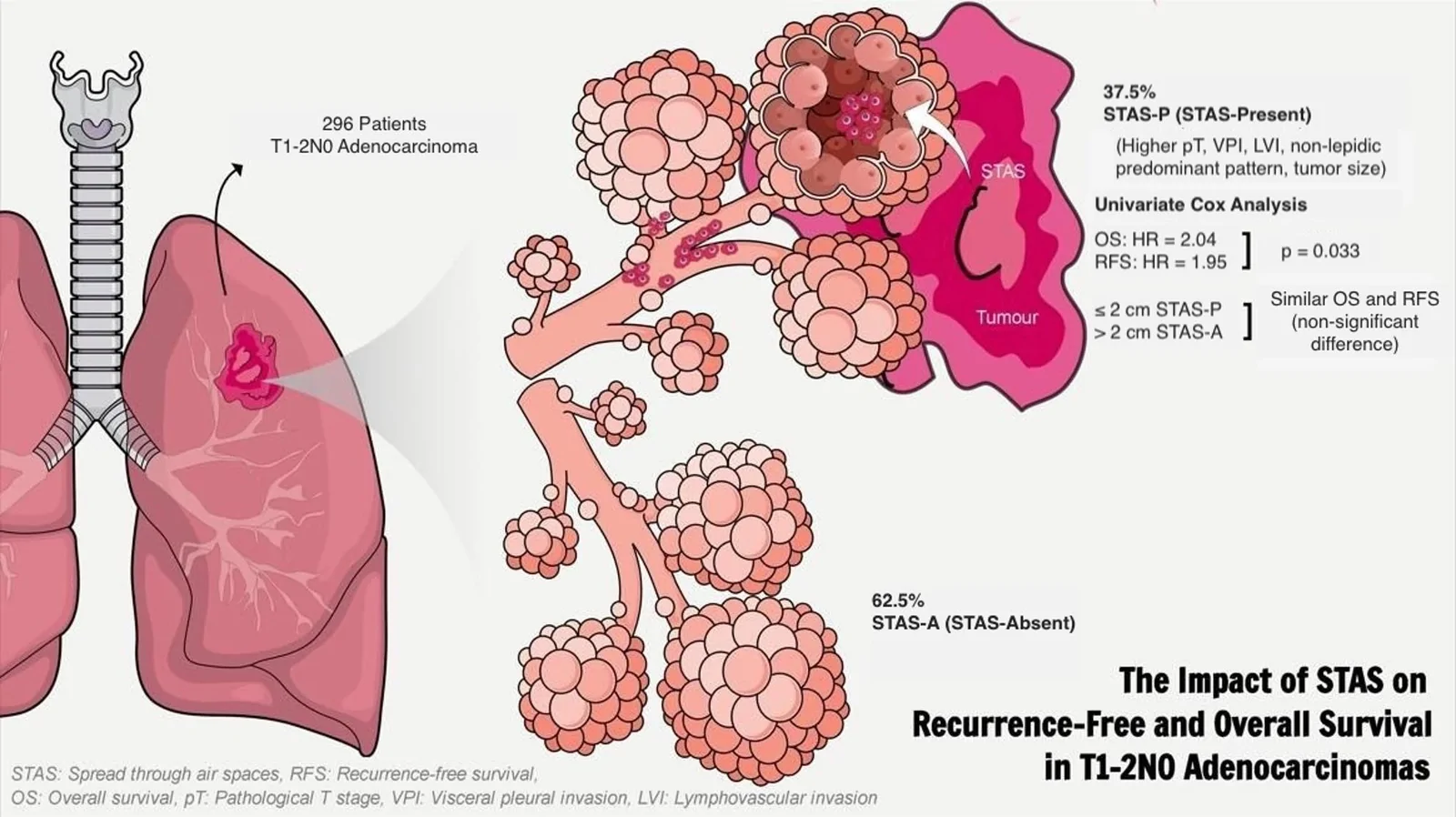

## Introduction

Lung cancer (LC) is the worldwide leading cause of cancer-related mortality, thus making it an important public health concern [1]. In the last decade, there were significant advances in the diagnosis and treatment of LC. Thanks to technological advances, we can detect very early-stage LCs, such as ground-glass nodules, with greater sensitivity [2]. Moreover, there is growing evidence supporting the effectiveness of LC screening programs and their impact on survival outcomes [3, 4]. 

As a result, the number of diagnosed early-stage LC has increased. The choice of surgical approach and the lung resection extent in these cases are vital for optimizing survival outcomes [5].

Complete resection is still the best treatment option for early-stage, node-negative lung adenocarcinoma (AC), which is the most common subtype of LC. However, recurrence occurs even in node-negative, surgically resected non-small cell lung cancer (NSCLC) patients. Despite advances in early detection and minimally invasive surgical techniques, a significant portion of patients with early-stage lung AC still experience recurrence and/or decreased survival [6]. Along with the conventionally accepted prognostic parameters such as tumor, node, metastasis (TNM) classification, histopathological subtype, visceral pleura invasion (VPI), lymphovascular invasion (LVI), and perineural invasion (PNI), the prognostic value of spread through air spaces (STAS) has attracted increasing attention, thus potentially making it a guide for personalized treatment strategies [7–10].

STAS, defined as the presence of tumor cells within the first alveolar spaces of the lung parenchyma beyond the edge of the main tumor, was first recognized in the 2015 World Health Organization (WHO) classification [11]. Since then, the interest in STAS has grown considerably, and its prognostic value is now increasingly recognized [7–10], despite ongoing debates about its incorporation into the 9th TNM classification [12]. Nevertheless, controversy remains regarding its precise definition, as well as reported differences in incidence and outcomes across histopathological subtypes and patient populations [12, 13]. Moreover, the extent to which STAS influences survival in early-stage lung AC remains incompletely defined.

The aim of this study was to evaluate the prognostic relevance of STAS and its association with pathological and clinical variables in a large, single-center surgically resected T1-2N0 cohort, with particular attention to how its prognostic impact interacts with tumor size at the ≤ 2 cm threshold relevant to sublobar resection decisions.

## Materials and methods

### Ethical approval

Ethical approval was obtained from the Ankara University Faculty of Medicine Human Research Ethics Committee (approval number İ07-681-25; September 7, 2025), and the study was conducted in accordance with the Declaration of Helsinki.

### Study population

A total of 986 patients had available STAS data after undergoing curative complete resection for NSCLC between 2016 and 2024 at the Department of Thoracic Surgery, Ankara University Faculty of Medicine. All of those retrospectively inspected patients underwent preoperative evaluations, which included routine laboratory tests and clinical staging using computed tomography (CT), 18 F-fluorodeoxyglucose positron emission tomography-CT, and brain imaging.

Patients were eligible for inclusion if they had pathologically confirmed lung AC with T1-2N0 disease according to the TNM classification and had undergone curative-intent complete surgical resection with available STAS data. Patients who received neoadjuvant treatment, those with distant metastasis and node-positive tumors, T3/4 tumors, or pathologies other than AC as indicated in their final pathology report were excluded from this study. After applying the inclusion and exclusion criteria, a total of 296 patients who had T1/2N0 lung AC were included in the final analysis. Patients who had poor prognostic features were given adjuvant chemotherapy according to the oncologist’s clinical decision.

### Data collection

Electronic medical records were used to gather clinical, surgical, and pathological data. Histopathological data included the assessment of STAS, tumor subtype, tumor size (mm), pathological T stage (pT), LVI, PNI, and VPI.

### Statistical analysis

Patients were initially categorized as STAS-Present (STAS-P) and STAS-Absent (STAS-A). Associations between STAS presence and categorical variables were evaluated using chi-square test and Fisher’s Exact Test when expected cell counts were less than 5. Continuous variables were compared using Mann–Whitney U test, as the data were not normally distributed. Survival outcomes, overall survival (OS) and recurrence-free survival (RFS), were analyzed using Kaplan-Meier curves and compared using the log-rank test. The median follow-up duration was estimated using the reverse Kaplan–Meier curves. The Cox proportional hazards model was used to calculate hazard ratios (HRs) for OS and RFS. Multivariable Cox regression was performed including tumor size category, surgical procedure, VPI, and predominant histopathological subtype as pre-specified covariates selected based on clinical relevance. The model was intentionally parsimonious given the limited number of outcome events. Subgroup analysis was conducted based on tumor size category (≤ 2 cm vs. > 2 cm, based on clinical relevance for surgical decision-making) and STAS presence (STAS-P vs. STAS-A) combined. Subgroup analyses were exploratory in nature and performed without correction for multiple comparisons. Accordingly, findings should be interpreted as hypothesis-generating rather than confirmatory. To formally test whether the prognostic effect of STAS presence differed by tumor size category, a Cox regression model incorporating STAS, categorical tumor size, and their interaction was fitted for both OS and RFS. Statistical significance was set at *p* < 0.05.

## Results

Among 296 patients with lung AC, 111 (37.50%) were STAS-P, and 199 (67.23%) underwent a lobar procedure. The median age was 66 years (interquartile range (IQR): 12) and the median tumor size was 18 mm (IQR: 13). Age did not differ significantly between the groups of STAS-P and STAS-A.

STAS presence was significantly associated with more aggressive histopathological features, including micropapillary/solid predominant subtypes, higher pT stage, and the presence of LVI and VPI (Table 1).

**Table 1:** Patients and tumor characteristics

Characteristic	Total (*n* = 296) (%)	STAS-Absent (*n* = 185) (62.5)	STAS-Present (*n* = 111) (37.5)	*P*-value
Age (years)				0.387
Median (IQR)	66 (12)	65 (12)	66 (12)	
Sex				0.006
Male	159 (53.7)	88 (47.6)	71 (64.0)	
Female	137 (46.3)	97 (52.4)	40 (36.0)	
Smoking History*				0.042
Never	78 (26.5)	56 (30.6)	22 (19.8)	
Former/current	216 (73.5)	127 (69.4)	89 (80.2)	
Surgical Procedure				0.032
Lobar	199 (67.2)	116 (62.7)	83 (74.8)	
Sublobar	97 (32.8)	69 (37.3)	28 (25.2)	
Tumor Size (mm)				< 0.001
Median (IQR)	18 (13)	16 (14)	20 (12)	
Tumor Size Category				< 0.001
≤ 2 cm	164 (55.4)	117 (63.2)	47 (42.3)	
> 2 cm	132 (44.6)	68 (36.8)	64 (57.7)	
Pathological T Stage				0.005
T1a	47 (15.9)	36 (19.5)	11 (9.9)	
T1b	117 (39.5)	81 (43.8)	36 (32.4)	
T1c	51 (17.2)	30 (16.2)	21 (18.9)	
T2a	70 (23.6)	33 (17.8)	37 (33.3)	
T2b	11 (3.7)	5 (2.7)	6 (5.4)	
Lymphovascular Invasion				< 0.001
Absent	257 (86.8)	175 (94.6)	82 (73.9)	
Present	39 (13.2)	10 (5.4)	29 (26.1)	
Perineural Invasion				0.632
Absent	292 (98.6)	183 (98.9)	109 (98.2)	
Present	4 (1.4)	2 (1.1)	2 (1.8)	
Visceral Pleural Invasion				0.028
Absent	238 (80.4)	156 (84.3)	82 (73.9)	
Present	58 (19.6)	29 (15.7)	29 (26.1)	
Predominant Histopathological Subtype				< 0.001
Lepidic	72 (24.3)	67 (36.2)	5 (4.5)	
Acinar/papillary	170 (57.4)	99 (53.5)	71 (64.0)	
Micropapillary/solid	54 (18.2)	19 (10.3)	35 (31.5)	
Adjuvant Chemotherapy**				0.026
No	258 (92.1)	167 (94.9)	91 (87.5)	
Yes	22 (7.9)	9 (5.1)	13 (12.5)	
Recurrence				0.108
Absent	255 (86.1)	164 (88.6)	91 (82.0)	
Present	41 (13.9)	21 (11.4)	20 (18.0)	
Survival Status				0.043
Alive	260 (87.8)	168 (90.8)	92 (82.9)	
Exitus	36 (12.2)	17 (9.2)	19 (17.1)	

Values are *n* (%) unless otherwise stated. Mann-Whitney U test used for age and tumor size; chi-square test for categorical variables. Fisher’s Exact Test was used for Perineural Invasion due to low expected cell counts. *n = 294 (2 missing); percentages calculated among patients with available data. **n = 280 (16 missing); percentages calculated among patients with available data

**Fig. 1:**
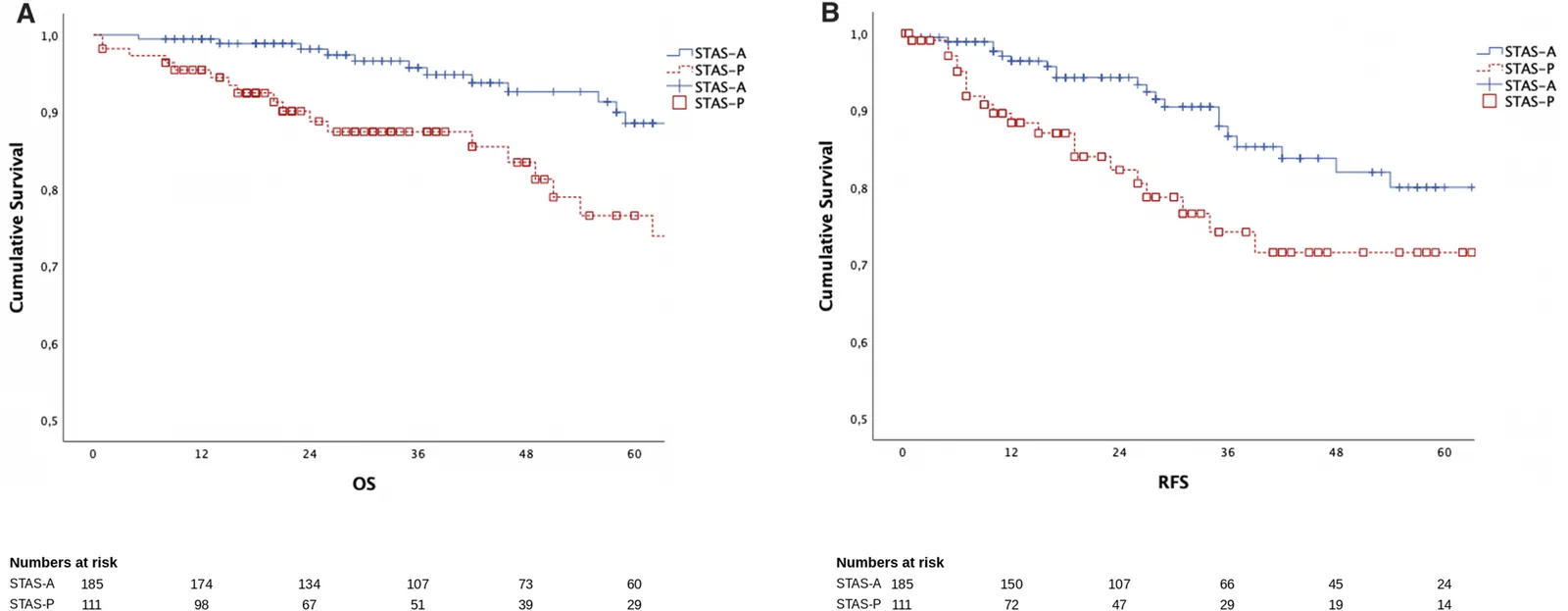
Kaplan-Meier curves for STAS status in OS (**A**) and RFS (**B**). Kaplan-Meier curves for OS (**A**) and RFS (**B**) by STAS status (log-rank p=0.030, for both). Numbers at risk at 0, 12, 24, 36, 48, and 60 months.

Among those 296 patients, recurrence was observed in 41 cases: 19 locoregional, 17 distant, and 5 involved both. During the follow-up period, a total of 36 patients died. The median follow-up duration was 41 months (95% confidence interval (CI): 36.1–45.9 months), estimated by the reverse Kaplan–Meier curves. The median OS and RFS were not reached in either group during the follow-up period, as fewer than 50% of patients experienced the respective events. The mean OS was 90.5 months (95% CI: 85.4–95.5) in the STAS-A group and 86.6 months (95% CI: 78.6–94.7) in the STAS-P group. The mean RFS was 76.8 months (95% CI: 71.5–82.1) in the STAS-A group and 77.0 months (95% CI: 68.1–85.9) in the STAS-P group. While the mean RFS values were similar, the Kaplan–Meier curves separated at later follow-up, a difference captured by the log-rank test but not reflected in the mean estimates. OS and RFS were significantly worse in the STAS-P group (*p* = 0.030 for both) using log-rank tests (Fig. 1; Table 2).

**Table 2:** Overall and pairwise univariate log-rank comparisons for STAS status and combined STAS status and tumor size subgroups in OS and RFS

Comparison	OS p-value	RFS p-value
Overall log-rank tests		
STAS-A vs STAS-P	0.030	0.030
STAS + Tumor size: 4-group overall test	0.026	0.011
Pairwise log-rank comparisons between STAS status and Tumor size subgroups		
STAS-A ≤2 cm vs STAS-P ≤2 cm*	0.041	0.092
STAS-A ≤2 cm vs STAS-A >2 cm	0.029	0.022
STAS-A ≤2 cm vs STAS-P >2 cm	0.001	0.001
STAS-A >2 cm vs STAS-P ≤2 cm*	0.916	0.705
STAS-A >2 cm vs STAS-P >2 cm*	0.446	0.308
STAS-P ≤2 cm vs STAS-P >2 cm	0.491	0.231

*Highlighted rows: (1) STAS-A ≤2 cm vs STAS-*P* ≤2 cm — STAS-presence significantly worsens prognosis within the small tumor group (OS *p* = 0.041); (2) STAS-A >2 cm vs STAS-*P* ≤2 cm — no significant difference, indicating STAS-*P* small tumors carry a prognosis equivalent to STAS-A larger tumors (OS *p* = 0.916, RFS *p* = 0.705); (3) STAS-A >2 cm vs STAS-*P* >2 cm — no significant difference, suggesting STAS adds limited prognostic discrimination when tumor size is already >2 cm (OS *p* = 0.446, RFS *p* = 0.308)

*OS* overall survival, *RFS* recurrence-free survival, *STAS-A* STAS-absent, *STAS-P* STAS-present

The univariate Cox proportional hazards model indicated that STAS may be considered a significant prognostic predictor of both OS (HR = 2.038, *p* = 0.033) and RFS (HR = 1.949, *p* = 0.033) (Table 3).

**Table 3:** The Univariate Cox proportional hazards model for STAS status and combined STAS status and Tumor size subgroups in OS and RFS

Variable	HR	95% CI	*p*
STAS status			
Overall Survival			
STAS-A	Reference	—	—
STAS-P	2.04	1.06–3.98	0.033
STAS + Tumor size* (overall test)	—	—	0.046
STAS-A ≤ 2 cm	Reference	—	—
STAS-A > 2 cm	3.25	1.14–9.23	0.027
STAS-*P* ≤ 2 cm	3.33	1.02–10.93	0.047
STAS-*P* > 2 cm	4.39	1.56–12.33	0.005
Recurrence-Free Survival			
STAS status			
STAS-A	Reference	—	—
STAS-P	1.95	1.06–3.60	0.033
STAS + Tumor size* (overall test)	—	—	0.002
STAS-A ≤ 2 cm	Reference	—	—
STAS-A > 2 cm	2.84	1.14–7.03	0.025
STAS-*P* ≤ 2 cm	2.36	0.79–7.03	0.123
STAS-*P* > 2 cm	4.23	1.71–10.49	0.002

*Overall omnibus Chi-square p-value for the STAS–tumor size 4-group categorical variable; reference category for subgroup comparisons is STAS-A ≤ 2 cm

*HR* Hazard ratio, *CI* Confidence interval, *STAS-A* STAS-absent, *STAS-P* STAS-present

In multivariable Cox analysis incorporating categorical tumor size, surgical procedure as either lobar or sublobar, VPI, and predominant histopathological subtype as covariates, STAS did not retain independent significance for either OS (HR = 1.87, 95% CI 0.90–3.88, *p* = 0.092) or RFS (HR = 1.583, 95% CI 0.82–3.05, *p* = 0.170). Surgical procedure emerged as the only independent predictor of both OS (HR = 2.89, 95% CI 1.45–5.77, *p* = 0.003) and RFS (HR = 4.08, 95% CI 2.09–7.98, *p* < 0.001). When a simplified model including only STAS presence and surgical procedure was fitted, both variables retained independent significance for both OS (STAS: HR = 2.33, 95% CI 1.19–4.53, *p* = 0.013; surgical procedure: HR = 2.23, 95% CI 1.14–4.35, *p* = 0.019) and RFS (STAS: HR = 2.31, 95% CI 1.23–4.33, *p* = 0.009; surgical procedure: HR = 2.47, 95% CI 1.31–4.67, *p* = 0.005).

In the subgroup analysis, patients were stratified in an exploratory manner by combining tumor size subgroups and STAS status:

- STAS-A + Tumor size ≤ 2 cm.
- STAS-A + Tumor size > 2 cm.
- STAS-P + Tumor size ≤ 2 cm.
- STAS-P + Tumor size > 2 cm.

**Fig. 2:**
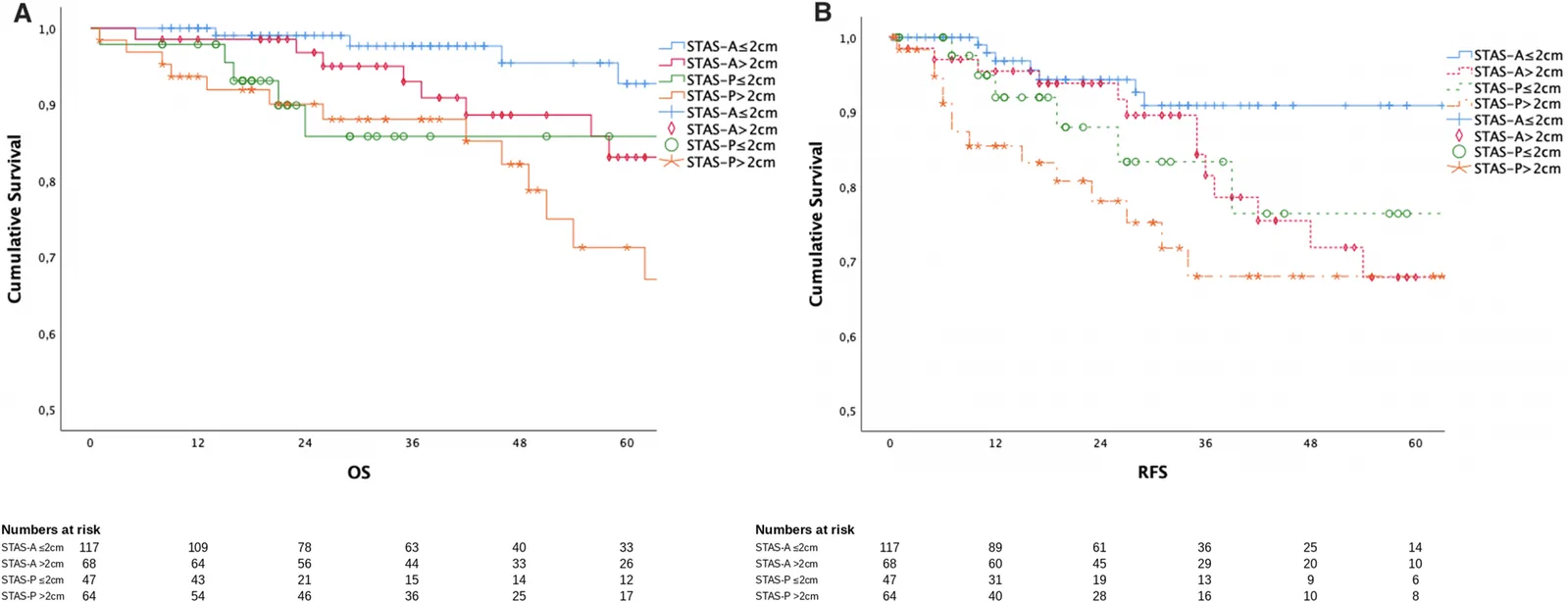
Kaplan-Meier curves for combined STAS status and tumor size subgroups in OS (**A**) and RFS (**B**). Kaplan-Meier curves for OS (**A**) and RFS (**B**) by combined STAS status and tumor size category (log-rank *p* = 0.026 and *p* = 0.011, respectively). Numbers at risk at 0, 12, 24, 36, 48, and 60 months

Kaplan-Meier curves showed a progressive decline in survival across these four groups (Fig. 2).

The Cox proportional hazards model indicated that, compared to the reference group (STAS-A ≤ 2 cm), STAS-*P* > 2 cm showed the worst outcomes as expected, with an OS HR of 4.39 (*p* = 0.005) and RFS HR of 4.23 (*p* = 0.002) (Table 3). Though, a formal interaction test between STAS status and tumor size category was not statistically significant for either OS (*p* = 0.214) or RFS (*p* = 0.495).

Notably, further analysis using the log-rank test and Kaplan-Meier curves indicated that there was no statistically significant difference in OS and RFS between patients with STAS-A > 2 cm and those with STAS-*P* ≤ 2 cm (*p* = 0.916 and *p* = 0.705, respectively) (Fig. 2; Table 2).

Adjuvant chemotherapy was administered more frequently in STAS-P than in STAS-A patients (12.5% vs. 5.1%, *p* = 0.026). To assess whether this imbalance influenced the survival findings, a sensitivity analysis was performed after excluding patients who received adjuvant chemotherapy (*n* = 22) and those with missing treatment status (*n* = 16), leaving 258 patients (167 STAS-A, 91 STAS-P).

In this subset, the STAS-associated difference in RFS remained significant (log-rank *p* = 0.008; univariate HR = 2.41, *p* = 0.011), whereas the OS difference did not reach significance (log-rank *p* = 0.121; univariate HR = 1.84, *p* = 0.128), with only 25 of the 36 death events remaining in this subset.

## Discussion

In this cohort of T1-2N0 lung adenocarcinoma, STAS was associated with worse OS and RFS on univariate analysis and with the features of aggressive tumor biology, although it did not remain an independent predictor of survival after multivariable adjustment. Consistent with the recent International Association for the Study of Lung Cancer (IASLC) recommendation to include STAS as a histopathological descriptor in the ninth edition of the TNM classification [12], these survival findings and associations are in line with its reported prognostic relevance [7–10]. Collectively, they suggest that STAS is not merely an incidental histopathological feature but a reflection of a more aggressive tumor biology. Additionally, the significantly worse OS observed even in an early-stage cohort with a relatively short median follow-up of 41 months may reflect the biological aggressiveness that STAS confers beyond local recurrence risk [14].

The aforementioned aggressive features were also evident in the histological distribution of STAS. Consistent with the existing literature, our findings showed that STAS was less frequently observed in the lepidic predominant subtype, which often presents as nodules with a ground-glass component.

STAS was significantly more prevalent in micropapillary and solid predominant subtypes, as well as in tumors with LVI and VPI, features indicating more aggressive tumor biology [7, 8]. Given that the lepidic predominant subtype is associated with more indolent biological behavior [11], the lower incidence of STAS in these cases is consistent with the use of parenchyma-sparing resections in selected patients [8, 15].

The absence of independent significance for STAS in multivariable analysis in our study after adjustment for categorical tumor size, VPI, histopathological subtype, and surgical procedure (either lobar or sublobar) is not unexpected and should be interpreted in context rather than as evidence against its prognostic relevance. STAS is not an isolated biological phenomenon; it co-exists with other features of aggressive tumor behavior as demonstrated in our cohort as well. This collinearity naturally attenuates its coefficient when these variables are simultaneously adjusted. Similar findings have been reported in other retrospective STAS series, where univariate significance was not consistently reproduced in multivariable models [7, 16]. Furthermore, when a simplified model including only STAS status and surgical procedure was fitted, both variables retained independent significance for both OS and RFS, suggesting that the attenuation in the full model largely reflects collinearity rather than an absence of prognostic value. The magnitude of the HR observed for surgical procedure in the full multivariable model likely reflects the same multicollinearity, as the covariates included are themselves associated with STAS presence. This underscores the interpretive caution warranted when applying multivariable Cox models in cohorts with strong intercorrelations among clinicopathological variables.

For the subgroup analysis, univariate pairwise log-rank test adds further depth to these observations.

Patients with STAS-P tumors > 2 cm had the worst survival outcomes, as expected. However, the absence of significant difference in survival outcomes between the patients with STAS-A tumors > 2 cm and those with STAS-P tumors ≤ 2 cm was particularly notable. The comparable survival of STAS-P tumors ≤ 2 cm and STAS-A tumors > 2 cm likely reflects the joint contribution of STAS and tumor size, rather than the independent effect of either alone. Depending solely on tumor size without considering STAS status may lead to underestimation of recurrence risk, especially in patients with small but biologically aggressive early-stage lung AC.

Pairwise univariate log-rank analysis further revealed that unlike previously reported findings suggesting STAS is more impactful in larger tumors [14], the prognostic impact of STAS appeared most pronounced in tumors ≤ 2 cm, where STAS-P tumors showed significantly worse OS compared to STAS-A counterparts of equivalent size, whereas no significant difference in OS and RFS were observed between STAS-P and STAS-A patients in the > 2 cm group (Table 2). As these pairwise comparisons were exploratory and uncorrected for multiple testing, and as formal interaction testing between STAS status and tumor size did not reach statistical significance for either OS or RFS, these subgroup findings should be interpreted as hypothesis-generating. Moreover, given the limited number of outcome events in our cohort, this interaction test was likely underpowered to detect a true effect modification, and a non-significant result should not be interpreted as definitive evidence against an interaction between STAS status and tumor size. This pattern suggests that in larger tumors, the size-related risk may already dominate the prognostic picture, making STAS a less discriminating additional factor. In contrast, among small tumors where size alone would predict a favorable outcome, STAS presence may overturn that expectation.

These observations may have clinical relevance in the context of the current trend towards sublobar resection for early-stage, node-negative tumors ≤ 2 cm [15]. Our data may suggest that STAS-P tumors ≤ 2 cm may represent a biologically distinct, higher-risk subgroup. Within this subgroup, STAS status may be relevant to risk stratification when planning the extent of resection. This raises the hypothesis that STAS status may warrant consideration in individualized surgical planning, pending validation. Prior studies have reported that lobectomy may be associated with better outcomes than sublobar resections in STAS-P tumors [8, 17]. However, our study did not stratify survival outcomes by extent of resection, and therefore cannot determine whether lobectomy confers a survival benefit over sublobar resections specifically in STAS-P patients. Prospective, adequately powered studies stratifying resection outcomes by STAS status are needed before firm surgical recommendations can be made.

Our findings raise the question of whether STAS assessment can be incorporated into intraoperative frozen section analysis to guide the extent of resection. Although theoretically attractive, the practical application is highly challenging, as STAS is often heterogeneously distributed and difficult to capture reliably in limited frozen sections; complete assessment currently requires examination of the whole specimen [13, 18–20]. Developing more accurate intraoperative techniques to identify STAS could therefore contribute to individualized surgical decision-making.

The presence of STAS, given its association with a higher recurrence risk, may necessitate closer follow-up and consideration of adjuvant therapy in affected patients [10, 21]. In our study, the potential confounding effect of adjuvant chemotherapy warrants consideration. Adjuvant treatment was administered at the oncologist’s decision based on adverse pathological features, and STAS-P patients received adjuvant therapy more frequently. This uneven distribution reflects selection bias inherent to retrospective design and may have partially attenuated the observed survival difference between groups rather than amplified it. As presented in the Results, a sensitivity analysis excluding adjuvant-treated and treatment-status-unknown patients confirmed that the STAS-associated RFS difference persisted, supporting that our primary findings were not driven by differential adjuvant treatment. The corresponding OS difference lost significance in this smaller subset, likely reflecting the substantially reduced number of death events available for analysis rather than a true absence of effect.

STAS is an increasingly recognized but still incompletely standardized invasive feature in lung adenocarcinoma [7–13]. STAS assessment remains subject to well-recognized pitfalls, including tumor floaters and artifacts from specimen handling, all of which must be excluded [12, 13, 22]. The subjective nature of this evaluation contributes to variable reported STAS incidence across different institutions, regions, and populations [12, 13]. This complicates consistent assessment in multi-center studies, underlining the need for consensus criteria and prospective validation with both global and region-specific studies [13]. Given that a significant number of patients still experience recurrence despite advances in detection and surgery, incorporating STAS status into risk assessment may improve stratification and inform personalized management, including the extent of resection, follow-up, and adjuvant treatment, despite the challenges in its assessment [10, 12, 21].

This study has several limitations. Its retrospective, single-center design limits generalizability and precludes causal inference. STAS was assessed by experienced thoracic pathologists using standardized criteria based on the 2015 WHO classification (11); however, formal interobserver reproducibility (e.g., kappa statistics) was not performed. The number of outcome events was limited, which constrains the power of multivariable analyses and subgroup comparisons, including the formal test for an interaction between STAS status and tumor size. Furthermore, the median follow-up of 41 months may be insufficient to fully capture the long-term prognostic impact of STAS, particularly for overall survival, given that late recurrences are not uncommon in early-stage lung AC. Additionally, the sensitivity analysis excluding adjuvant-treated and treatment-status-unknown patients was likely underpowered for OS given the reduced number of events. Sublobar resections were not further classified into segmentectomy and wedge resection, as the primary aim was to evaluate the prognostic impact of STAS rather than to compare surgical approaches. Moreover, our data do not permit conclusions about the optimal surgical approach in STAS-positive patients, as the limited number of events precluded stratified survival analysis by extent of resection; this question requires adequately powered, ideally multi-center or pooled, studies with procedure-specific stratification. Similarly, the number of recurrence events was insufficient to meaningfully compare locoregional versus distant recurrence patterns by STAS status.

## Conclusion

In early-stage lung AC, STAS was a significant prognostic factor on univariate analysis, although it did not retain independent significance after multivariable adjustment. In the exploratory subgroup analysis, STAS-P tumors ≤ 2 cm carried a survival burden comparable to that of STAS-A tumors > 2 cm, a hypothesis-generating finding that warrants prospective validation before informing surgical management. Routine STAS assessment in resected specimens may nonetheless be clinically informative, given its association with other adverse histopathological features.

## Data Availability

The datasets used and/or analysed during the current study are available from the corresponding author on reasonable request.

## Abbreviations

AC: Adenocarcinoma
CI: Confidence Interval
CT: Computed Tomography
HR: Hazard Ratio
IASLC: International Association for the Study of Lung Cancer
IQR: Interquartile Range
LC: Lung Cancer
LVI: Lymphovascular Invasion
NSCLC: Non-Small Cell Lung Cancer
OS: Overall Survival
PNI: Perineural Invasion
pT: Pathological T Stage
RFS: Recurrence-Free Survival
STAS: Spread Through Air Spaces
STAS-A: STAS-Absent
STAS-P: STAS-Present
TNM: Tumor, Node, Metastasis
VPI: Visceral Pleura Invasion
WHO: World Health Organization
